# Functional significance of ion channels during macropinosome resolution in immune cells

**DOI:** 10.3389/fphys.2022.1037758

**Published:** 2022-10-20

**Authors:** Masashi Maekawa, Ren Natsume, Makoto Arita

**Affiliations:** ^1^ Division of Physiological Chemistry and Metabolism, Graduate School of Pharmaceutical Sciences, Keio University, Tokyo, Japan; ^2^ Laboratory for Metabolomics, RIKEN Center for Integrative Medical Sciences (IMS), Yokohama, Kanagawa, Japan; ^3^ Cellular and Molecular Epigenetics Laboratory, Graduate School of Medical Life Science, Yokohama-City University, Yokohama, Kanagawa, Japan

**Keywords:** TMEM206, macropinocytosis, macropinosome resolution, two-pore channel (TPC), macrophages

## Abstract

Macropinocytosis is a unique type of endocytosis accompanied by membrane ruffle formation. Closure of membrane ruffles leads to the uptake of large volumes of fluid phase and, subsequently, the formation of large vacuoles termed macropinosomes. Immune cells, such as dendritic cells, T cells, and macrophages, endocytose the surrounding amino acids and pathogens *via* macropinocytosis either constitutively or in a stimulus-dependent fashion. This process is critical for cell migration, mammalian target of rapamycin complex 1 (mTORC1) activation, and antigen presentation. Large vacuoles are fragmented into tubules and smaller vesicles during the progression and maturation of macropinosomes in immune cells. This process is called “macropinosome resolution” and requires osmotically driven shrinkage of macropinosomes, which is controlled by ion channels present in them. The crenation of membranes on shrunken macropinosomes is recognized by curvature-sensing proteins and results in intracellular membrane trafficking. In this mini review, we highlight the recent progress in research on macropinosome resolution in macrophages, with a focus on ion channels (TPC1/2 for Na^+^ and TMEM206 for Cl^−^) that is required for macropinosome resolution. We also discuss the potential contribution of membrane lipids to this process.

## 1 Introduction

### 1.1 Physiological roles of macropinocytosis in immune cells

**FIGURE 1:**
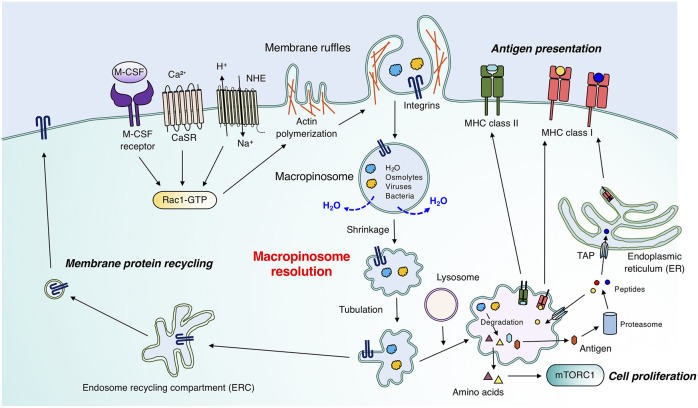
Physiological roles of macropinocytosis in immune cells. Immature dendritic cells, macrophages, and T cells constitutively and/or stimulus-dependently form membrane ruffles enriched with F-actin. Signal transduction involving CasR and the M-CSF receptor enables membrane ruffle formation through Rac1 activation. Macropinosomes are generated through membrane ruffle closure, which requires a proper spatio-temporal phosphoinositide metabolism pattern. Large vacuoles follow two main fates during macropinosome maturation. The first is the degradation pathway by fusion with lysosomes, which leads to the degradation of extracellular substances such as antigens and cell debris. In T cells and macrophages, amino acids obtained through macropinocytosis can activate mTORC1 for efficient cell proliferation. Especially in immature dendritic cells, degraded antigens are transported to the plasma membrane for antigen presentation by MHC class I or II. MHC class II and degraded antigens form complexes in matured macropinosomes, which were fused with lysosomes, and follow the vesicular pathway. Antigens passed through the cytosol are subjected to proteasomal degradation, and degraded peptides are transported into the endoplasmic reticulum (ER) or matured macropinosomes through “transporter associated with antigen processing” (TAP), followed by recognition by MHC class I. The second pathway is the recycling pathway to the plasma membrane. Adhesion molecules, such as integrins, on macropinosomes are recycled back to the plasma membrane, which is critical for immune cell migration. Macropinosomes shrink through osmotically driven water loss in the recycling pathway (see also Figure 2A).

Among a variety of endocytic pathways, macropinocytosis plays pivotal physiological roles in immune cells (Figure 1). Macropinocytosis is the uptake of large amounts of liquid phase accompanied by dynamic morphological changes in the plasma membrane, leading to the formation of membrane ruffles (Swanson, 2008). The membrane ruffles then fuse to form macropinosomes (0.2–10 μm in diameter), which are large vesicles containing extracellular particles and fluid (Swanson, 2008). This unique type of endocytosis was first documented in macrophages by Warren Lewis in the 1930s (Lewis, 1937). To date, a variety of physiological roles of macropinocytosis in immune cells have been reported.

#### 1.1.1 Immature dendritic cells

Immature dendritic cells actively and non-selectively endocytose large amounts of antigens from the extracellular fluid *via* macropinocytosis and degrade the material in lysosomes, thereby leading to antigen presentation on the major histocompatibility complex (MHC) class II molecule (Sallusto et al., 1995; Garrett et al., 2000). Regarding cell migration, immature primary dendritic cells form macropinosomes at the cell front to sense hydraulic resistance. This leads to efficient extracellular space exploration (Moreau et al., 2019), which emphasizes the significance of macropinocytosis for the immune surveillance function in immature dendritic cells.

#### 1.1.2 Macrophages

The MHC class I presentation by macrophage colony stimulating factor (M-CSF)-induced bone marrow-derived macrophages (BMDMs) is inhibited by the Na^+^/H^+^ exchanger (NHE) inhibitor, amiloride (Norbury et al., 1997). Given the necessity of NHEs in macropinocytosis (Koivusalo et al., 2010), the above-mentioned study indicates the contribution of this process to cross-presentation (described in Section 2). Macrophages endocytose extracellular proteins through macropinocytosis, followed by their lysosomal degradation; the degraded proteins are utilized to construct cellular components (Toh et al., 2019). In anti-inflammatory (M2-type) macrophages, macropinocytosis is constitutively active *via* sensing of extracellular Ca^2+^ (Canton et al., 2016). The constitutive macropinocytosis is more active in anti-inflammatory macrophages than in proinflammatory macrophages. Macropinocytosis in proinflammatory macrophages can be induced by various agonists of phosphatidylinositol-3 kinase (PI3K) such as cytokines and chemokines (Redka et al., 2018). The differences in macropinocytic activities between anti-inflammatory and proinflammatory macrophages are likely derived from the divergent activities of PI3K and Rho-GTPases (Redka et al., 2018). The antigen-presenting capacity of anti-inflammatory macrophages is greater than that of proinflammatory macrophages (Redka et al., 2018). Further studies are required to reveal the physiological meanings of macropinocytosis as a means of antigen presentation by macrophages.

#### 1.1.3 Neutrophils and T cells

Neutrophils can perform macropinocytosis for the uptake of insoluble immune complexes and graphene oxide-based biomaterials (Lu et al., 2020; Karmakar et al., 2021). Similar to immature dendritic cells, neutrophils are likely to select migration paths with less hydraulic resistance in a phenomenon known as barotaxis (Prentice-Mott et al., 2013), which could be mediated by macropinocytosis. Macropinocytosis is also essential to sustain mTORC1 activation in CD4^+^ T cells by the uptake of amino acids, leading to efficient T cell proliferation (Charpentier et al., 2020). The macropinocytosed materials in CD4^+^ T cells are transported to lysosomes, leading to the phosphorylation of p70 S6 kinase, which is a hallmark of mTORC activation (Charpentier et al., 2020).

### 1.2 Pathological roles of macropinocytosis

From a pathological standpoint, a variety of viruses (e.g., vaccinia virus and Ebola virus) and bacteria (e.g., *Salmonella* and *Shigella*) invade and infect host cells by utilizing macropinocytosis (Francis et al., 1993; Nanbo et al., 2010; Mercer and Helenius, 2012; Weiner et al., 2016). Although macropinocytosis activities in host cells such as epithelial cells are normally low, *Salmonella* and *Shigella* induce macropinocytosis of host cells through effectors that are injected into the cytosol *via* a type III secretion system, in which the relevant effectors have yet to be determined (Francis et al., 1993; Chen et al., 1996; Cossart and Helenius, 2014; Weiner et al., 2016). These studies suggest the pathological significance of macropinocytosis in immune cells.

### 1.3 Macropinosome resolution in immune cells

Although the molecular mechanisms underlying membrane ruffle formation and its closure have been well characterized, with a focus on actin polymerization and membrane lipid metabolism, respectively (Levin et al., 2015), the maturation process of macropinosomes in the cytosol is not well understood. The nascent macropinosomes are fused with various endosomes and changes their characteristics followed by travelling the degradation or recycling routes. This overall process is called macropinosome maturation. Recently, high-resolution live-cell imaging has been used to show that macropinosomes dynamically change their morphology during their trafficking in immune cells (Freeman et al., 2020). This process in which the volume of macropinosomes is decreased and membrane fragmentation is occurred during their maturation is called “macropinosome resolution” (Freeman et al., 2020). Macropinosome resolution requires parallel efflux of ions and osmotically coupled water. In macrophages, Na^+^ and Cl^−^ are transported from macropinosomes into the cytosol by specific ion channels (Freeman et al., 2020; Zeziulia et al., 2022). The altered shape of the macropinosome is then recognized by the effector proteins and leads to the formation of tubules and small vesicles that transport cargos to the target membranes. In this mini review, we summarize the molecular mechanisms of macropinocytosis while focusing on macropinosome resolution, which is a process of “water loss from macropinosomes” in macrophages and is regulated by ion channels present in the macropinosomes.

## 2 Mechanism of macropinocytosis in immune cells

The process of macropinocytosis can be divided into three steps—1) membrane ruffle formation, 2) membrane ruffle closure, and 3) macropinosome maturation. The formation of membrane ruffles from periphery and dorsal areas of the plasma membrane requires Rac1- and Cdc42-triggered p21 activated kinase 1 (PAK-1)-dependent actin rearrangement (Ridley et al., 1992; Dharmawardhane et al., 2000; Koivusalo et al., 2010). Amiloride, an NHE inhibitor, suppresses macropinocytosis by causing proximal acidification beneath the plasma membrane, which impairs the localization and activation of Rac1 and Cdc42 (Koivusalo et al., 2010). A G-protein-coupled calcium sensing receptor (CaSR) senses extracellular Ca^2+^, leading to Rac1 and/or Cdc42 activation, which is followed by membrane ruffle formation during constitutively active macropinocytosis in anti-inflammatory macrophages and immature dendritic cells (Canton et al., 2016).

In macrophages, the dynamic spatio-temporal metabolic pattern of phosphoinositides, a class of lipids, at the cytosolic leaflets of membrane ruffles is crucial during membrane ruffle closure, which precedes macropinosome formation (Levin et al., 2015; Swanson and Araki, 2022). Phosphatidylinositol-3,4,5 trisphosphate [PI(3,4,5)P_3_] is generated by class I PI3K (heterodimers of p110/p85 or p110/p101) from phosphatidylinositol-4,5 bisphosphate [PI(4,5)P_2_] at the membrane ruffles (Araki et al., 1996; Araki et al., 2003). The generation of PI(3,4,5)P_3_ coordinates actin depolymerization (Levin et al., 2015). The treatment of macrophages with class I PI3K inhibitors inhibits the formation of membrane ruffle closures (Araki et al., 1996; Araki et al., 2003), which suggests that PI(3,4,5)P_3_ is essential for this process. Although class I PI3 kinases are important for the fusogenic step in macropinosome closure, the coordination of lipids and/or other machineries remains to be elucidated (Quinn et al., 2021).

After formation of macropinosomes, several regulators of membrane trafficking, such as Rab and sorting nexin (SNX) proteins (Rab5, Rab8a, Rab10, Rab20, Rab21, SNX1, SNX5), localize to the macropinosomes during maturation in macrophages (Egami and Araki, 2009; Egami and Araki, 2012; Lim et al., 2012; Welliver and Swanson, 2012; Wall et al., 2019; Liu et al., 2020; Kawai et al., 2021). The depletion of SNX5 reduced both size and number of macropinosomes in BMDMs (Lim et al., 2012). Rab8a on the macropinosomes is essential for Akt phosphorylation in lipopolysaccharide (LPS)-stimulated mouse RAW264.7 macrophages (Wall et al., 2019). However, molecular mechanisms underlying the recruitment of these effector proteins on macropinosomes are still unclear.

## 3 Ion channels and their osmoregulation of macropinosomes in immune cells

### 3.1 Endosomal ion dynamics by ion channels

Although all types of pinocytosis involve the uptake of fluid, the fate of endosomal fluid derived from extracellular milieu remains unclear (Saric and Freeman, 2020). Cells endocytose the extracellular fluid through various pinocytic pathways; however, the cell volume is constant during pinocytosis (Steinman et al., 1976; Chadwick et al., 2021). This suggests the existence of osmolarity-driven water efflux from endosome to the cytosol and then to the extracellular space. The major ions responsible for the osmolarity in cells are monovalent inorganic ions, including Na^+^, K^+^, and Cl^−^ (Chadwick et al., 2021). The concentrations of Na^+^ and Cl^−^ (110–140 mM) in the extracellular fluid are 3–10 times higher than those in the cytosol (Morgan et al., 2011; Scott and Gruenberg, 2011). In contrast, the cytosolic concentration of K^+^ ion is much higher (140 mM) than its extracellular concentration of 4 mM (Steinberg et al., 2010; Saric and Freeman, 2020). In principle, ion composition of the fluid phase inside nascent endosomes just after scission from the plasma membrane during pinocytosis should be identical to that of the extracellular fluid. Like other large vacuoles, macropinosomes are quasi-spherical: early steps in macropinosome maturation involving fast recycling of membrane only serve to further minimize their surface: volume. Promptly, the comparatively minute forces to remove macropinosomal surfaces may be offset by the hydrostatic pressure and high tensions on the limiting membranes of macripinosomes. To relieve this tension, concentration gradients of ions such as Na^+^ and Cl^−^ generate a mechanism of water loss, decreasing hydrostatic pressure, and ultimately attenuating membrane tension of macropinosomes. In early endosomes, Na^+^ is transported into the cytosol through lipid-gated monovalent cation channels, two pore channels (TPCs), and transient receptor potential mucolipins (TRPMLs) (Chen et al., 2021). Chloride channels (ClCs), as well as volume-regulated anion channel (VRAC), can potentially facilitate Cl^−^ efflux into the cytosol in early endosomes (Argenzio and Moolenaar, 2016; Jentsch, 2016; Jentsch and Pusch, 2018). ClCs transports two Cl^−^ ions into the cytosol, accompanied by influx of one H^+^ ion in early endosomes, and NHEs allow for one Na^+^ ion to be transported in exchange for one H^+^ ion. This finding suggests that ClCs and NHEs contribute to acidification of early endosomes (Kondapalli et al., 2014; Saric and Freeman, 2020). Hypothetically, various ion channels and exchangers could facilitate the macropinosome shrinkage. Since ion channels are more efficient in ion efflux/influx than exchangers, ion channels would be more critical in the osmotic regulation of macripinosomes than exchangers.

### 3.2 Involvement of Na^+^ and Cl^−^ ion channels in macropinosome resolution

Ion exchange and water efflux affect the volume of macropinosomes. Macropinosomes are much larger than other vesicles generated in other pinocytic pathways, such as clathrin-mediated and caveolae-mediated endocytosis. A simple question in the process of macropinosome maturation is whether the size of macropinosomes is maintained throughout maturation. In 2020, Freeman et al. (2020) addressed this by analyzing the temporal changes in macropinosome volumes using diffraction-limited microscopy. They found that BMDMs formed large macropinosomes (average volume 7 μm^3^) within 5 min of M-CSF stimulation, together with an increase in cell volume (Freeman et al., 2020). The volume of the macropinosome decreased over time, and the cell volume was restored to the basal level 30 min after M-CSF stimulation (Freeman et al., 2020). It was further confirmed that ion substitutions, such as N-methyl-D-glucamine^+^ for Na^+^ and gluconate^−^ for Cl^−^, remarkably inhibited macropinosome shrinkage in BMDMs. This suggests that efflux of particularly Na^+^ and Cl^−^ ions from macropinosomes to the cytosol is essential for macropinosome resolution (Freeman et al., 2020). This re-affirms that the volume control of macropinosomes is osmotically coupled. Interestingly, luminal Ca^2+^ ions in macropinosomes are not involved in this process (Freeman et al., 2020). These observations suggest the existence of macropinosomal Na^+^ and Cl^−^ ion channels that are essential for macropinosome resolution.

### 3.3 Macropinosome resolution regulated by two-pore channels

**FIGURE 2:**
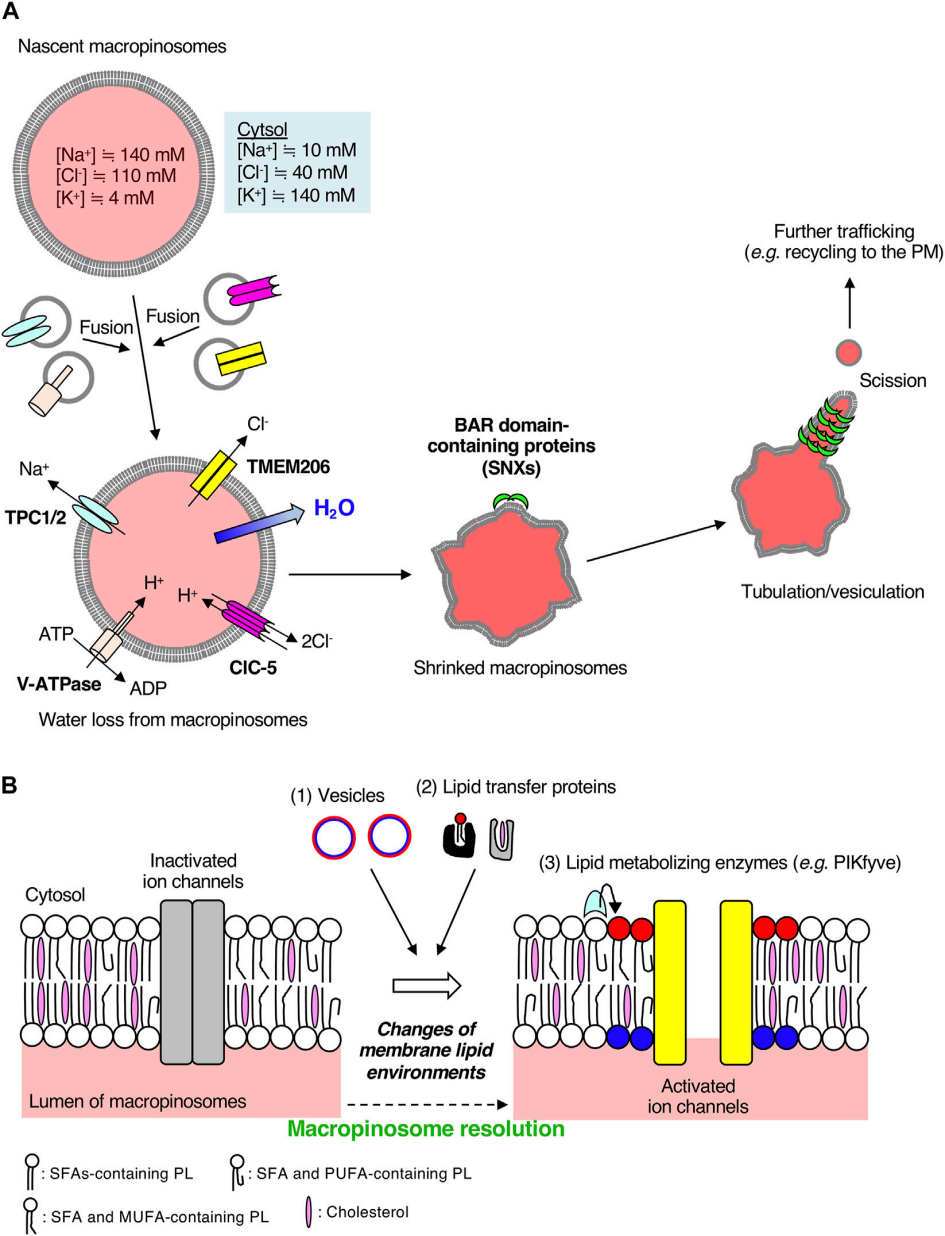
Macropinosomal ion channels export Na^+^ and Cl^−^ into the cytosol during macropinosome resolution. **(A)** A scheme of macropinosome resolution in BMDMs. The ion concentrations inside nascent macropinosomes match extracellular ion concentrations. Osmotically driven ion exchange occurs through macropinosomal ion channels along with water efflux into the cytosol after the fusion of endosomes on which a variety of ion channels are localized. TMEM206 is activated by depolarization of macropinosomal membranes mediated by Na^+^ efflux through TPCs and luminal acidification by V-ATPases and CLC 2Cl^−^/H^+^ exchangers. Loss of water from macropinosomes facilitates crenation, which is recognized by BAR-domain containing proteins followed by tubulation, vesiculation, and further membrane trafficking. The color inside macropinosomes represents the concentration of TMR-dextran. **(B)** Possible involvement of the lipid environments of macropinosomal membranes to ion channel activities. Ion channels on cellular membranes are regulated by their surrounding lipid environments. The lipid composition of macropinosomal membranes could be changed during the process of macropinosome resolution. Lipids can be supplied through (1) membrane trafficking and (2) lipid transfer proteins. Polar head groups of phospholipids such as phosphoinositides can be enzymatically converted to other phospholipid species (3). For example, a membrane phospholipid PI(3,5)P_2_, which is generated by PIKfyve from PI3P, activates TPCs on macropinosomes (Freeman et al., 2020). Molecular structures of fatty acids vary (e.g., saturated fatty acids, monounsaturated fatty acids, polyunsaturated fatty acids), and hence, the fatty acid composition of phospholipids could also affect ion channels and membrane curvature. Cholesterol content affects membrane fluidity and lipid nanodomains. Elucidation of lipid composition of macropinosomal membranes is required for further understanding of the resolution mechanisms. SFA, saturated fatty acid; MUFA, monounsaturated fatty acid; PUFA, polyunsaturated fatty acid; PL, phospholipid. Red and blue circles represent phospholipids in the cytosolic and luminal leaflets of the vesicles, respectively. After fusion with target macropinosomes, phospholipids shown in red and blue are located in the cytosolic and luminal leaflets of macropinosomal membranes, respectively.

TPCs are voltage-gated selective cation channels (Wang et al., 2012; She et al., 2018) and are involved in a variety of pathophysiological events, such as development, metabolism, and tumor formation, through the regulation of Ca^2+^ signaling (Jin et al., 2020). TPC1 localizes to a range of endosomes and TPC2 localizes to late endosomes and lysosomes (Ruas et al., 2014). Knockout of TPC1/2 leads to prolonged activation of EGFR signaling on endolysosomes (Müller et al., 2021). During the process of macropinosome maturation, treatment of BMDMs with a TPC inhibitor, tetrandrine, suppresses the reduction in macropinosome volume, which is similar to that observed for BMDMs from TPC1/TPC2 double-knockout mice (Freeman et al., 2020). These two TPC channels are functionally redundant and localize to macropinosome (Freeman et al., 2020). Collectively, TPC1 and TPC2 are the macropinosomal Na^+^ channels responsible for macropinosome resolution in macrophages (Figure 2A). TPC channels are activated by NAADP and phosphatidylinositol-3,5 bisphosphate [PI(3,5)P_2_] leading to Ca^2+^ and Na^+^ efflux from endosomes to the cytosol, respectively (Patel et al., 2022). Since luminal Ca^2+^ ions in macropinosomes are not required for macropinosome resolution (Freeman et al., 2020), PI(3,5)P_2_ on macropinosomal membrane may activate TPC channels.

### 3.4 Macropinosome resolution regulated by TMEM206

The outward conductance of Na^+^ alone would be electrogenic, and create a membrane-potential that could tend to oppose the process. Thus, the anion outward conductance (such as Cl^−^) is required. Recently, some studies have identified TMEM206 as a Cl^−^ ion channel essential for macropinosome shrinkage in macrophages (Ullrich et al., 2019; Yang et al., 2019; Zeziulia et al., 2022). TMEM206 was originally identified as a component of the acid-sensitive outwardly rectifying (ASOR) anion channel (Ullrich et al., 2019). Although the depletion of TMEM206 protects HEK293 cells from acid-induced death (Ullrich et al., 2019), the pathophysiological roles of TMEM206 still remain unclear. Among various Cl^−^ ion channels, TMEM206 and ClC-7 are highly expressed in BMDMs and are mainly localized on early endosomes and lysosomes, respectively (Zeziulia et al., 2022). During macropinosome maturation, TMEM206 localizes to the macropinosome in BMDMs and is likely supplied from EEA1- or Rab5-positive early endosomes (Zeziulia et al., 2022). As were seen in TPC1/TPC2 double-knockout macrophages, macropinosome resolution was impaired in BMDMs derived from TMEM206 knockout mice (Zeziulia et al., 2022). These observations suggest that TMEM206 is the macropinosomal Cl^−^ channel responsible for macropinosome resolution in macrophages (Figure 2A).

### 3.5 Orchestration of macropinosmal ion channels for macropinosome resolution

The ion channel activity of TMEM206 is controlled by other ion channels (Figure 2A). Since TMEM206 is activated by Na^+^ influx-mediated membrane depolarization (Ullrich et al., 2019), TPCs-mediated Na^+^ transport from macropinosomes to the cytosol causes the TMEM206 activation. TMEM206 forms a trimeric chloride channel and is activated by protons (Deng et al., 2021). Thus, luminal acidification of macropinosomes leads to the activation of TMEM206 followed by macropinosome resolution. In BMDMs, the luminal alkalinization by treatment with NH_4_Cl abolishes macropinosome resolution (Zeziulia et al., 2022), suggesting the pH dependency for TMEM206-mediated Cl^−^ efflux from macropinosomes. ClC-5 is an exchanger of Cl^−^ and H^+^, which is important for the early acidification of endosomes in epithelial cells (Scheel et al., 2005; Novarino et al., 2010). Expression of a mutant version of ClC-5, which mediates the export of two Cl^−^ ions from endosomes to the cytosol in a voltage-independent manner accompanied by the import of one H^+^ into endosomes, restores the defects of macropinosome resolution in TMEM206 knockout BMDMs (Picollo and Pusch, 2005; Zeziulia et al., 2022). ClC-5 is another macropinosome resident Cl^−^ ion channel that colocalizes with TMEM206 in BMDMs (Zeziulia et al., 2022). Taken together, luminal acidification of macropinosomes by V-ATPases and ClC Cl^−^/H^+^ exchangers (mainly ClC-5) contributes to the TMEM206-mediated Cl^−^ exit from macropinosomes to the cytosol (Figure 2A) (Zeziulia et al., 2022).

### 3.6 Physiological importance of macropinosome resolution in macrophages

Osmotically driven shrinkage of macropinosomes by efflux of Na^+^ and Cl^−^ ions remarkably changes the morphology of macropinosomes. Since macropinosome shrinkage causes the crenation of macropinosomal membranes, high membrane curvatures from the lumen to the cytosol are formed on the macropinosome surface. Then, BAR domain-containing proteins, such as SNX1, SNX2, and SNX5, recognize the membrane curvature, leading to the tubulation from macropinosomes and membrane recycling (Figure 2A) (Cullen, 2008; Freeman et al., 2020). Membrane tubulation plays pivotal roles in various pathways of membrane trafficking, including recycling of membrane proteins to the plasma membrane (Figure 2A) (Grant and Donaldson, 2009). In fact, inhibition of TPC channels reduces the plasmalemmal localization of Mac-1 (integrin αMβ2, a key molecule to macrophage adherence and migration) and results in the accumulation of endosomal integrin β1 in BMDMs (Freeman et al., 2020). Critically, *in vivo*, administration of a TPC channel inhibitor in mice suppresses both the macropinosome resolution and the ability of immune surveillance (migration area/time) of macrophages in the peritoneal serosa (Freeman et al., 2020). Thus, osmotically driven macropinosome resolution, as well as the resolution of fluid, through the efflux of Na^+^ is necessary for macropinosome tubulation in macrophages, followed by membrane protein recycling, which leads to optimal immune cell function *in vivo*. Anti-inflammatory macrophages are migratory, and macropinosome formation is critical for seeking the migration pathways through barotaxis (Moreau et al., 2019; Lennon-Duménil and Moreau, 2021). Thus, macropinosome resolution at the cell front in anti-inflammatory macrophages may reduce the sensitivity to hydraulic resistance, contributing to their migration capacity.

## 4 Perspective

In this mini review, we summarized the critical roles of ion channels (TPCs for Na^+^ and TMEM206 for Cl^−^) localized on macropinosomes in regulating the osmolarity of large vacuoles, ultimately leading to the resolution of their luminal fluid and resorption of their membranes in BMDMs. Since neighboring membrane lipid environments of ion channels affect their activities, dynamic changes in the lipid composition of macropinosome could regulate macropinosomal ion channel activities (Figure 2B). Generally, lipid composition in organelles is determined by membrane trafficking, lipid transfer proteins, and local enzymes (Figure 2B). The lipid composition of macropinosomes has not been elucidated to date. Application of untargeted lipidomics for isolated macropinosomes can help in elucidating their unique lipid composition during maturation/resolution (Tsugawa et al., 2020; Stévenin et al., 2021). The lipidomics analysis would provide us with the fatty acid composition of macropinosomal phospholipids (Figure 2B). The saturated fatty acids-containing phospholipids forms liquid-ordered domains. As the degree of unsaturation in fatty acids increases, the membrane phospholipids form loosened domains. Thus, the fatty acid composition of macropinosomal phospholipids and cholesterol content in macropinosomal membranes may also be key regulators of ion channel activities because they control membrane fluidity and curvatures. TRPML2, an endo-lysosomal cation channel, is evoked by both hypotonic stimulation and mechanical force stimulus (Chen et al., 2020), suggesting the possibility of regulation of ion channels by membrane curvature. Spatio-temporal dynamics of lipid localization on macropinosomes in living cells can be monitored by making use of lipid visualizing probes (Hammond et al., 2022). The details of lipid composition of macropinosomes should be investigated in future studies.
